# MiR-378a suppresses tenogenic differentiation and tendon repair by targeting at TGF-β2

**DOI:** 10.1186/s13287-019-1216-y

**Published:** 2019-03-29

**Authors:** Yang Liu, Lu Feng, Jia Xu, Zhengmeng Yang, Tianyi Wu, Jiajun Zhang, Liu Shi, Dahai Zhu, Jinfang Zhang, Gang Li

**Affiliations:** 1 Department of Orthopaedics and Traumatology, Faculty of Medicine, Prince of Wales Hospital, The Chinese University of Hong Kong, Hong Kong, China; 2 Department of Molecular Medicine and Gene Therapy, Faculty of Medicine, Lund University, Lund, Sweden; 3 Department of Orthopaedic Surgery, Shanghai Jiao Tong University Affiliated Sixth People’s Hospital, Shanghai, China; 4 The State Key Laboratory of Medical Molecular Biology, Institute of Basic Medical Sciences, Chinese Academy of Medical Sciences and School of Basic Medicine, Peking Union Medical College, Beijing, China; 5 Key Laboratory of Orthopaedics and Traumatology, The First Affiliated Hospital of Guangzhou University of Chinese Medicine, The First Clinical Medical College, Guangzhou University of Chinese Medicine, Guangzhou, China; 6 Lingnan Medical Research Center, Guangzhou University of Chinese Medicine, Guangzhou, China; 7 Li Ka Shing Institute of Health Sciences, Prince of Wales Hospital, The Chinese University of Hong Kong, Shatin, Hong Kong, China

## Abstract

**Background:**

Tendons are a crucial component of the musculoskeletal system and responsible for transmission forces derived from muscle to bone. Patients with tendon injuries are often observed with decreased collagen production and matrix degeneration, and healing of tendon injuries remains a challenge as a result of limited understanding of tendon biology. Recent studies highlight the contribution of miR-378a on the regulation gene expression during tendon differentiation.

**Methods:**

We examined the tendon microstructure and tendon repair with using miR-378a knock-in transgenic mice, and the tendon-derived stem cells were also isolated from transgenic mice to study their tenogenic differentiation ability. Meanwhile, the expression levels of tenogenic markers were also examined in mouse tendon-derived stem cells transfected with miR-378a mimics during tenogenic differentiation. With using online prediction software and luciferase reporter assay, the binding target of miR-378a was also studied.

**Results:**

Our results indicated miR-378a impairs tenogenic differentiation and tendon repair by inhibition collagen and extracellular matrix production both in vitro and in vivo. We also demonstrated that miR-378a exert its inhibitory role during tenogenic differentiation through binding at TGFβ2 by luciferase reporter assay and western blot.

**Conclusions:**

Our investigation suggests that miR-378a could be considered as a new potential biomarker for tendon injury diagnosis or drug target for a possible therapeutic approach in future clinical practice.

**Electronic supplementary material:**

The online version of this article (10.1186/s13287-019-1216-y) contains supplementary material, which is available to authorized users.

## Background

Tendons are a crucial component of the musculoskeletal system and responsible for the transmission forces derived from muscle to bone. Tendon injury, such as tendon rupture and tendinopathy, is the common disease especially in athletes and the aging population [1, 2]. The injured tendons are always accompanied with altered collagen production, impaired extracellular matrix remodeling, and inflammatory cell infiltration, which lead to the incidence of re-injury [3]. Currently, clinical treatments for tendon injuries are limited to the injection of nonsteroidal anti-inflammatory drugs, physical therapy, or surgery, which are limited to pain relief [4, 5]. Therefore, a better understanding of the tendon pathology is essential for developing a new potential biomarker or drug target for possible therapeutic approach in clinical practice.

Endogenous microRNAs (miRNAs) are small noncoding RNA molecules with an average length of ~ 22 nucleotides [6]. They negatively regulate the target gene expression by binding the 3′-untranslated region (UTR) of their target mRNA leading to translational repression or mRNA degradation [7]. Several miRNAs have been shown to make contribution to tendon injuries [8]. For example, miRNA-29a was shown to regulate tissue remodeling as well as improve the treatment during early tendon injury [9, 10]. Recently, Thankam et al. showed that miR-378a was one of the most highly altered miRNAs in patients with rotator cuff tears, which indicated it may play a regulatory role during tendon injuries [8]. The two mature strands, miR-378a-3p and miR-378-5p, originate from the first intron of the peroxisome proliferate-activated receptor gamma, coactivator 1 beta (*ppargc1b*) gene encoding PGC-1β [11]. Embedding in the transcriptional regulator of oxidative energy metabolism gene implies its involvement in metabolic pathways and mitochondrial energy homeostasis, such as muscle development, differentiation, and regeneration [12]. Recent studies also reported the regulation of miR-378 in *MyoD* in muscle development and differentiation, indicating its essential biological role [13, 14]. However, it remains unknown how miR-378a exert its biological role during tendon differentiation and regeneration.

In our study, we would like to examine the role of miR-378a-3p (miR-378a) during tenogenic differentiation and tendon injury healing. Our results showed that tendon healing was inhibited by suppressing collagen and extracellular matrix (ECM) production in miR-378a knock-in transgenic mice, and miR-378a inhibits tenogenic differentiation in vitro. Using the prediction databases, miR-378a was shown to have the binding target at TGFβ2. We further confirmed protein expression of TGFβ2 was decreased in tendon-derived stem cells (TDSCs) from miR-378a knock-in mice, indicating that miR-378a inhibits tenogenic differentiation through binding at TGFβ2. Our investigation suggests that miR-378a could be considered as a new potential biomarker for tendon injury diagnosis or drug target for a possible therapeutic approach in clinical practice.

## Methods

### Mice

All animal studies were approved by the Animal Experimentation Ethics Committee of The Chinese University of Hong Kong. The generation of miR-378a knock-in transgenic (Tg) mice was according to a previous study [15]. The wild-type (WT) littermates (C57BL/6) were regarded as the control group. Six- to eight-week-old female mice were used in the study. Animals were maintained in a temperature-controlled room with a 12-h light/dark cycle and access to food and water ad libitum in the Laboratory Animal Service Center.

### TDSC isolation and cell culture

Mouse tendon-derived stem cells (TDSCs) were isolated from WT and Tg mice as previously described [16]. The cells were cultured in low-glucose DMEM (LG-DMEM) supplemented with 10% FBS, 100 U/ml penicillin, and 100 mg/ml streptomycin (all from Thermo Fisher Scientific, USA), at 37 °C with 5% CO_2_. For tenogenic differentiation, the TDSCs were incubated with complete LG-DMEM supplemented with 5 ng/ml TGFβ1 (PeproTech, USA). The cells were used at passages 4–6, and the medium was changed every 3 days.

### Cell transfection

The mouse miR-378a mimic (5′-ACUGGACUUGGAGUCAGAAGG-3′) and its negative control (NC, 5′-UUCUCCGAACGUGUCACGUTT-3′) and the inhibitor of miR-378a (anti-miR-378a, 5′-CCUUCUGACUCCAAGUCCAGU-3′) and its negative control (anti-NC, 5′-CAGUACUUUUGUGUAGUACAA-3′) were synthesized and purchased from GenePharma (Shanghai, China) and were transfected with Lipofectamine 3000 (Thermo Fisher Scientific, USA), according to the manufacturer’s protocol.

### Sirius Red staining

Collagen content was evaluated by Sirius Red staining and colorimetric assay. Briefly, TDSCs from WT and Tg were plated at 5000 cells/cm^2^ in a six-well plate and cultured in completed LG-DMEM. When cells became confluent, the culture medium was removed and cells were washed three times with PBS and were fixed with 4% paraformaldehyde for 10 min. One milliliter of saturated picric acid solution with 0.1% Sirius Red F3BA was added and incubated with shaking for 30 min at room temperature. The plate was washed with distilled water three times. To elute the color, 1 ml of 1:1 (*v*/*v*) 0.1% NaOH and absolute methanol was added to the plate, measured by using a spectrophotometer (BioTek Instruments, USA) at 540 nm to quantification of red color.

### Western blotting

The western blotting was performed as previously described [16]. Briefly, the total protein was isolated by RIPA lysis buffer supplemented with proteinase inhibitor (Sigma-Aldrich, USA). The protein was separated by SDS-PAGE (10%) and transferred to PVDF membranes (Bio-Rad, Hercules, CA, USA). After they were blocked with 5% skimmed milk for 1 h, the membranes were probed with the primary antibody to TGF-β2 (Abcam, USA) or GAPDH (EMD Millipore, USA). The membrane was incubated with horseradish peroxidase (HRP)-conjugated donkey anti-rabbit secondary antibody (Abcam, USA) after a wash with Tris-buffered saline containing Tween. The ECL substrate was used for developing the blot. GAPDH was used as internal control. ImageJ (NIH, USA) was used to quantify the intensity of each band as shown by ratio (relative to GAPDH).

### Tendon defect model

The tendon defect model was performed on 8-week-old male WT and Tg mice as previously described (*n* = 6 in each group at each time point) [16]. Briefly, the central part of the patellar tendon (PT) was removed without damaging the surrounding fibrocartilage region. The injured patellar tendon was collected for the histology study at week 2 and week 4 after the surgery. For sample collection, all the mice were euthanized, and the patellar tendon and surrounding joints were harvested for further ex vivo examination.

### Luciferase plasmid construction

For TGFβ2 luciferase reporter construction, the 400-bp fragments of the mouse TGFβ2 3′UTR coding region containing the predicted binding site were inserted into the pmir-GLO reporter vector to generate the TGFβ2-3′UTR-Wt Luciferase reporter. The binding site mutant vector was then generated by the Site-Directed Mutagenesis kit (Thermo Fisher Scientific, USA).

### Luciferase reporter assays

293T cells were used for the luciferase assays. The luciferase reporter plasmids were co-transfected with NC or miR-378a mimics. After incubation for 48 h, the cells were harvested. Firefly luciferase activities were measured with a Luciferase Assay System (Promega, USA) and normalized to Renilla luciferase activity, according to the manufacturer’s protocol.

### RNA isolation and real-time quantitative reverse transcription polymerase chain reaction (qRT-PCR)

Total RNA was isolated by using Trizol (Thermo Fisher Scientific, USA) from TDSCs of WT and Tg mice (*n* = 4) as previous described [16]. For mRNAs, they were reverse transcribed with using the PrimeScript RT reagent Kit (Takara Bio, Japan), followed by qRT-PCR with SYBR Green PCR Master Mix (Applied Biosystems, USA). For miRNAs, the reverse transcription and qRT-PCR were performed with using miRCURY LNA miRNA PCR kit (Qiagen, USA). All reactions were performed in triplicate. The primer sequences are shown in Additional file 1: Table S1. The miR-103a-3p was used as the internal control for analyzing miR-378a gene expression, and *Rpl13a*, *Ywhaz*, and *Gapdh* were regarded as the internal control for other genes as indicated.

### ELISA of protein concentrations in cell lysates

The protein contents of TDSCs treated with the miR-378a and anti-miR-378a mimics were measured by ELISA (Shanghai LanPai Biotechnology, China). The protein contents of TGFβ2 in cell lysates were analyzed as indicated in “Results”. The cell lysates were collected by RIPA lysis buffer supplemented with a proteinase inhibitor (Sigma-Aldrich, USA) and then analyzed by ELISA according to the manufacturer’s instructions. The concentration of total protein in each well was also measured and served as the internal control. The relative expression level of each protein is shown in “Results”.

### Histology analysis and immunohistochemistry staining

The isolated mouse patellar tendon and surrounding joint were fixed with 10% neutral-buffered formalin overnight (*n* = 6 of each group). Tissues were decalcified in 10% EDTA for 1 week, dehydrated, embedded in paraffin, cut into 5-mm-thick sections, and mounted on 3-aminopropyl-triethoxy-saline-coated slides (Sigma-Aldrich, USA). The sections were then stained with hematoxylin and eosin (H&E) for histological examination according to an established protocol [16]. Stained sections were then examined by light microscopy (Leica Microsystems, Germany). To count the cell number in the defect region, the regions of interests (1 μm × 1 μm) were located within the proximal, central, and distal parts of the defect region respectively. In each group, two sections from every mouse were examined.

For immunohistochemistry, the slides were rinsed in xylene to remove the paraffin, followed by rehydration in a graded series of ethanol. For blocking, 1% bovine serum albumin in PBS was applied for 30 min at room temperature. The sections were incubated with anti-Collagen type I (Abcam, USA) and anti-Thrombospondin 4 (Santa Cruz Biotechnology, USA) in a moist chamber overnight at 4 °C and washed in normal PBS the next day. Donkey anti-rabbit HRP-conjugated secondary antibodies (Abcam, USA) were added for an hour, followed by 3,39-diaminobenzidine tetrahydrochloride (Dako, Denmark) in the presence of H_2_O_2_. Afterward, the sections were rinsed, counterstained in hematoxylin, dehydrated with graded ethanol and xylene, mounted with DPX Permount (Sigma-Aldrich, USA), and examined by a microscope (Leica Microsystems, Germany). To do the semi-quantitative analysis, the percentage of the stained area relative to the total area under microscope view was measured using ImageJ [17]. In each group, four sections (four microscope views in each section) from every mouse were examined.

### Scanning electron microscopy (SEM)

Achilles tendons from WT and Tg mice (*n* = 4 in each group) were isolated and fixed in 2.5% glutaraldehyde and 0.05 M sodium phosphate buffer (pH 7.2) overnight. Samples were trimmed to a size of 1(H)× 1(W)× 2(L) mm, keeping the middle part of the tendon, and surrounding parts were removed the next day. Samples were then washed in 0.1 M cacodylate buffer and post fixation with 2% osmium tetroxide in 0.1 M cacodylate buffer. After fixation, samples were washed with 0.1 M cacodylate buffer, followed by dehydration via an ethanol series and critical point-drying with liquid CO_2_. The dried specimens were mounted on metal stubs and observed under SEM (Hitachi SU8010 Scanning Electron Microscope with iXRF EDS System, Tokyo, Japan).

### Transmission electron microscopy (TEM)

TEM was performed as previously reported [16]. Briefly, tendons from WT and Tg mice (*n* = 4) were isolated and fixed in 2.5% glutaraldehyde and 0.05 M sodium phosphate buffer (pH 7.2) overnight. Samples were trimmed to a size of 1 × 1 × 2 mm (length), keeping the middle part of tendon, and surrounding parts were removed the next day. Samples were then washed in 0.1 M cacodylate buffer and rinsed with physiologic saline, followed by dehydration via an ethanol series. Samples were embedded in epoxy resin and cut into ultrathin sections (∼ 50–100 nm). Sections were mounted on copper grids, contrasted with aqueous uranyl acetate and lead citrate, and examined. Images were recorded by a transmission electron microscope (H7700 Transmission Electron Microscope; Hitachi, Japan). Collagen fibril diameters and interfibrillar space were measured by using ImageJ software (NIH, USA), with five sections from each animal in both the WT and Tg groups.

### Statistical analysis

Data are presented as means ± SD. Student’s *t* tests were applied to compare between groups. All statistical analyses were performed by using SPSS Statistics (SPSS, Chicago, USA). Values of *p* < 0.05 were considered statistically significant.

## Results

### Impaired tendon tissues and delayed tendon injury healing were observed in miR-378a Tg mice

**Fig. 1:**
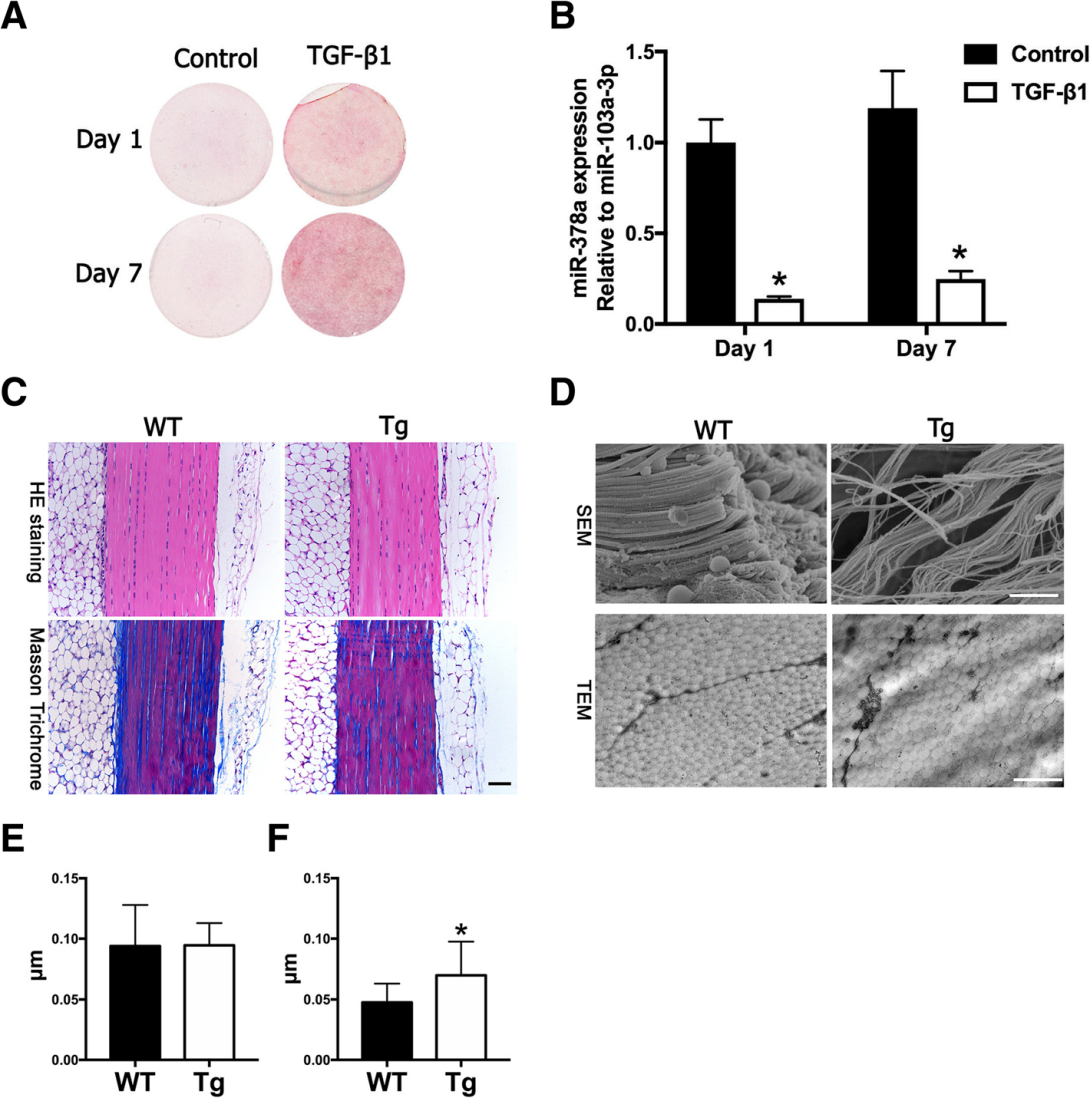
MiR-378a was inhibited during tenogenic differentiation with TGFβ1 and tendon tissue examination in miR-378 Tg mice. **a** Sirius Red staining of TDSCs under tenogenic differentiation with TGFβ1 (5 ng/ml) on day 1 and day 7 (*n* = 3; *p < 0.05 by Student’s *t* test). **b** The expression of mir-378a was inhibited during tenogenic differentiation on day 1 and day 7. **c** H&E and Masson Trichrome staining of tendon tissue in WT and Tg mice (scale bar = 200 μm). **d** Microstructure of tendon tissue in WT and Tg mice was examined by SEM and TEM (scale bar = 1 μm). **e** Quantification of diameters of tendon fibrils (*n* = 4). **f** Increased interfibrillar space between tendon fibrils in Tg mice compared with that in WT mice (*n* = 4, *p < 0.05 by Student’s *t* test)

In this study, 5 ng/ml TGFβ1 was applied to induce tenogenic differentiation, and our results showed that the collagen production was increased by TGFβ1 at day 1 and day 7, indicating that TGFβ1 successfully induced tenogenic differentiation of TDSCs (Fig. 1a). Importantly, miR-378a was decreased significantly at day 1 and day 7 during tenogenic differentiation (Fig. 1b), suggesting its negative function in tenogenic differentiation.

**Fig. 2:**
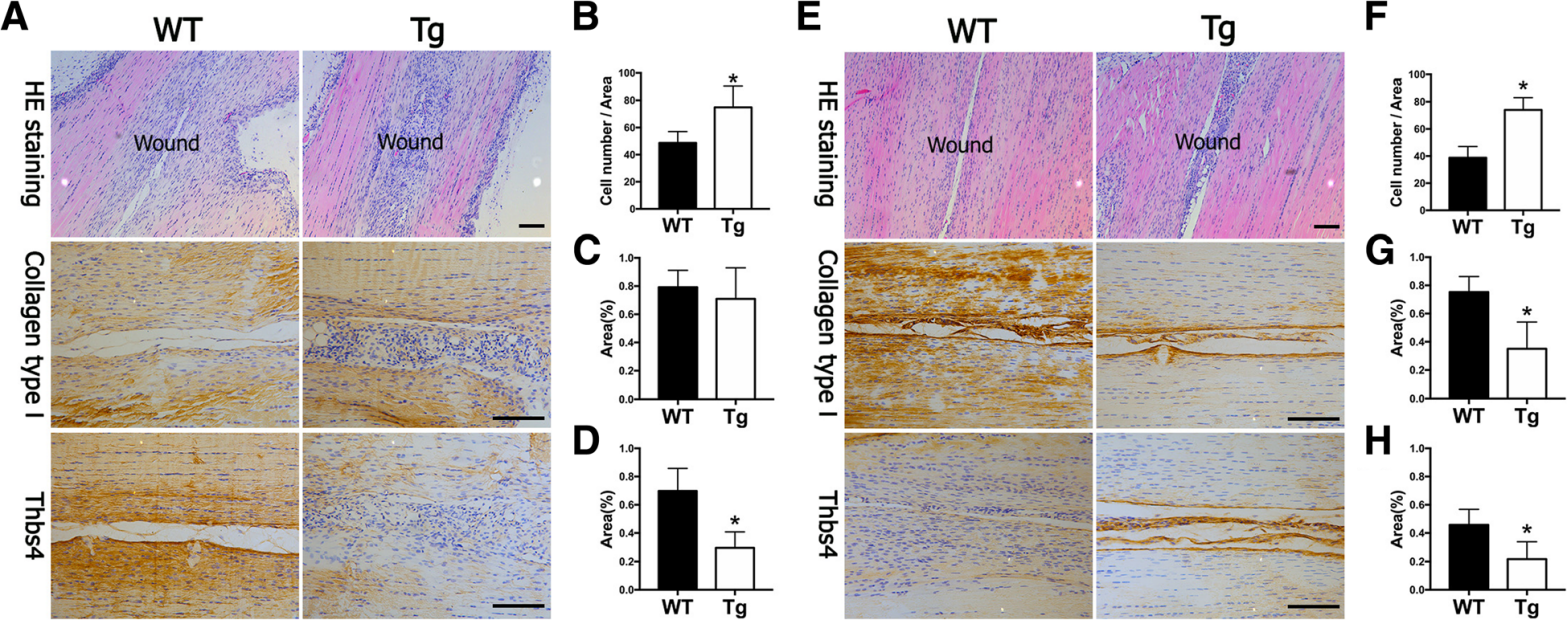
Tendon injury healing was impaired in miR-378a Tg mice. **a** H&E and immunohistochemistry staining (Collagen type 1 and Thbs4) of tendon defect in WT and Tg mice at week 2. **b** Increased cell numbers in defect regions of Tg mice at week 2 (*n* = 6, *, *p* < 0.05 by Student’s *t* test). **c**, **d** Semi-quantification of Collagen type I and Thbs4 expression area in the defect region at week 2 (*n* = 6, *, *p* < 0.05 by Student’s *t* test). **e** H&E and immunohistochemistry staining (Collagen type 1 and Thbs4) of tendon defect in WT and Tg mice at week 4. **f** Increased cell numbers in defect regions of Tg mice at week 4 (*n* = 6, *, *p* < 0.05 by Student’s *t* test). **g**, **h** Semi-quantification of the Collagen type 1 and Thbs4 expression area in the defect region at week 4 (*n* = 6, *p < 0.05 by Student’s *t* test). Scale bar = 100 μm

To further identify the function of miR-378a in tendon, a miR-378a transgenic (Tg) mouse was introduced. Tendon tissues from these Tg mice and those from wild-type (WT) mice were compared (Fig. 1c). H&E staining showed no significant difference in the tendon tissues between the two groups. The Masson Trichrome staining also showed evenly fiber organization between WT and Tg mice. By SEM examination, tendon fibrils exhibited loosely organized and irregular arrangements in Tg mice (Fig. 1d). To quantify the diameters of the tendon fibril and interfibrillar space, TEM was also performed. There was no significant difference on the diameters (Fig. 1e) and size distribution (Additional file 2: Figure S1) between the two groups, while the interfibrillar space in the Tg mice was significantly higher than that in the WT mice (Fig. 1f). To compare the tendon healing between WT and Tg mice, an established mouse tendon defect model was performed, and healing effects were examined at week 2 and week 4. As shown in Fig. 2, the wound region in Tg mice showed increased cell number (Fig. 2a, b) and decreased collagen type 1 and Thbs4 production at week 2 (Fig. 2c, d). At 4 weeks after injury, increased matrix production can be found in the defect region of the WT mice (Fig. 2e), while the Tg mice showed decreased matrix production accompanied with an increased cell number (Fig. 2f). And the production of collagen type 1 and Thbs4 was lower in the Tg mice compared with that in the WT mice (Fig. 2g, h). These results indicated that increased interfibrillar space can be observed in tendons from the Tg mice, and tendon injury healing was also inhibited with decreased collagen and ECM production in the Tg mice.

### Tenogenic differentiation was suppressed in TDSCs isolated from miR-378a transgenic mice

**Fig. 3:**
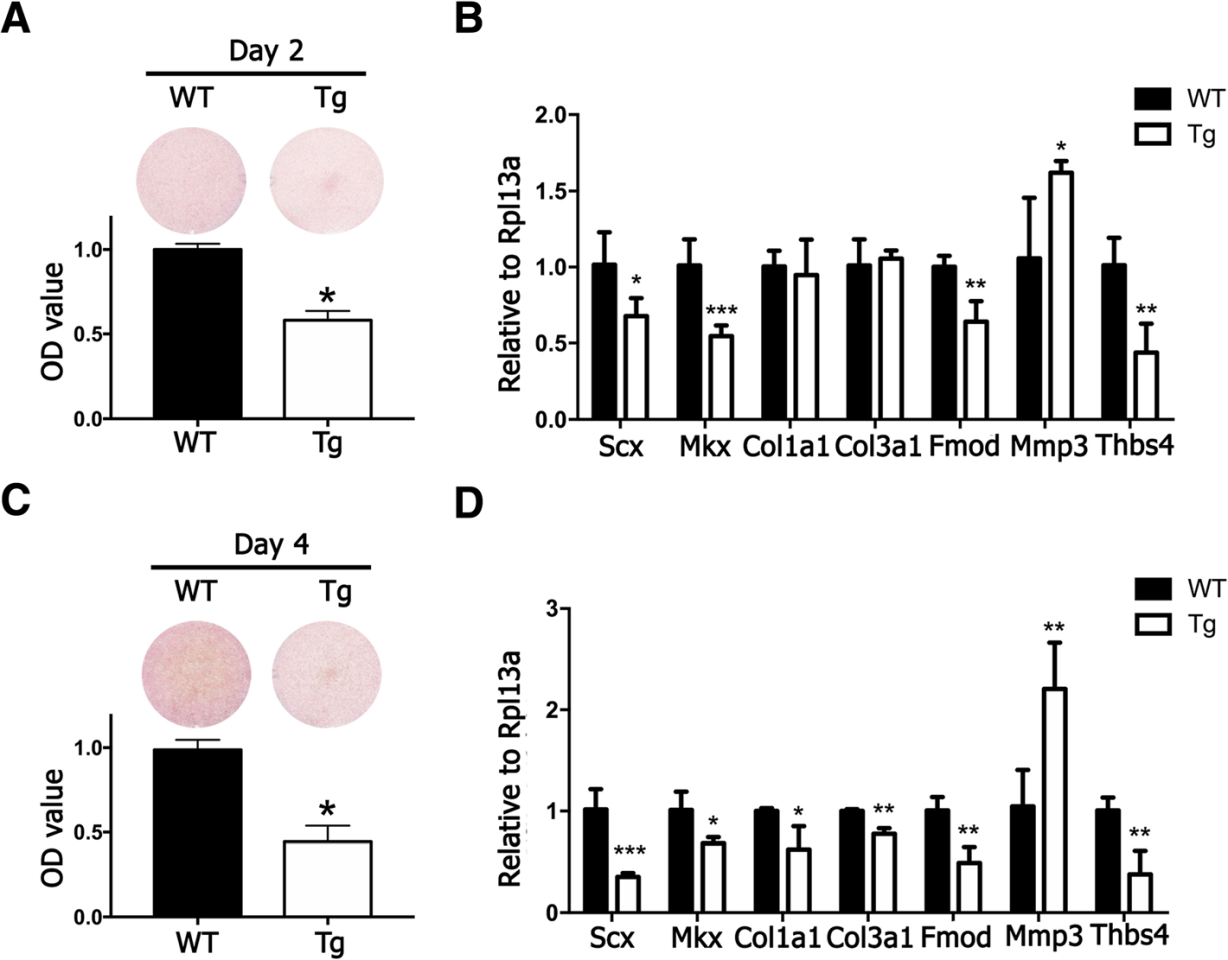
Tenogenic differentiation was suppressed in TDSCs derived from miR-378a Tg mice. **a** Sirius Red staining and OD value measurement at day 2. **b** Tenogenic marker expression at day 2. **c** Sirius Red staining and OD value measurement at day 4. **d** Tenogenic marker expression at day 4. *n* = 3; *p < 0.05 by Student’s *t* test; **p < 0.01 by Student’s *t* test; ***p < 0.001 by Student’s *t* test

To investigate the effect of miR-378a on tenogenic differentiation, TDSCs were isolated from WT and miR-378a Tg mice and their tenogenic differentiation potential was examined. As shown in Fig. 3a, c, decreased collagen production can be observed in TDSCs isolated from the Tg mice compared with that from the WT mice on day 2 and day 4 respectively. By comparing tenogenic marker expression, transcription factors such as *Scx* and *Mkx* were significantly decreased in TDSCs from the Tg mice compared with those from the WT mice on day 2 and day 4 respectively (Fig. 3b, d). The expression of collagen type 3 significantly decreased on day 2 and day 4, and collagen type 1 decreased significantly on day 4 in TDSCs from the Tg mice compared with those from the WT mice, which is consistence with Sirius Red results. On ECM marker expression, TDSCs from the Tg mice showed significantly decreased *Fmod* and *Thbs4* and increased *Mmp3* expression when compared with TDSCs from the WT mice on day 2 and day 4. Particularly, as *Ywhaz* gene was also shown being the most stable housekeeping gene during tenogenic induction in addition to *Rpl13a*, we also selected *Ywhaz* gene as an internal control when analyzing our data. The results with using *Ywhaz* as the internal control is shown in Additional file 3: Figure S2A & B, and the data remained consistent with *Rpl13a* as the internal control. Overall, our results further confirm the inhibitory role of miR-378a on tenogenic differentiation through suppressing collagen and ECM production.

### MiR-378a mimics inhibited tenogenic differentiation of TDSCs

**Fig. 4:**
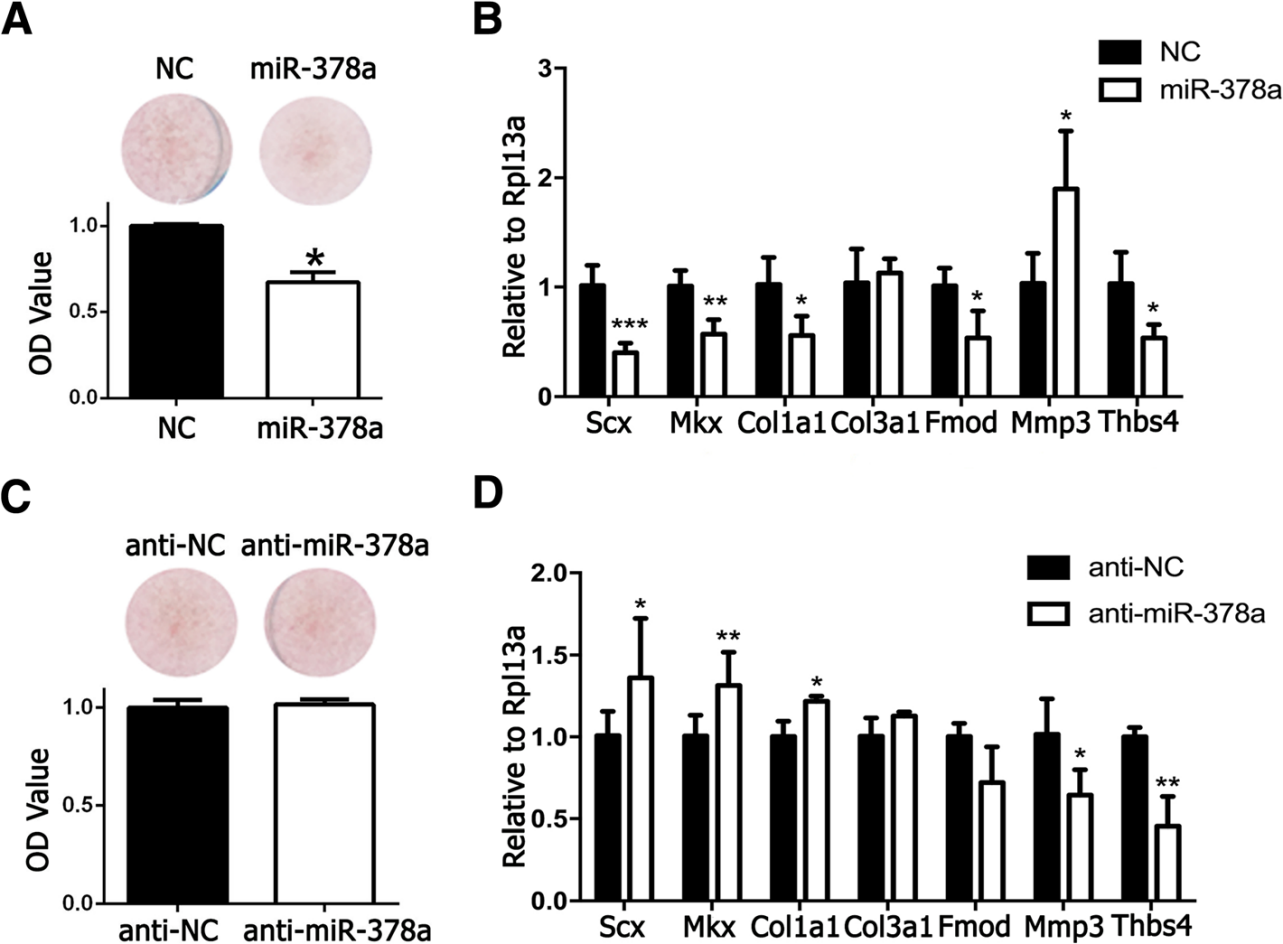
miR-378a mimics suppressed tenogenic differentiation of TDSCs. **a** Sirius Red staining and OD value of TDSCs transfected with miR-378a. **b** Tenogenic marker expression in TDSCs transfected with miR-378a. **c** Sirius Red staining and OD value of TDSCs transfected with anti-miR-378a. **d** Tenogenic marker expression in TDSCs transfected with anti-miR-378a. *n* = 3; *p < 0.05 by Student’s *t* test; **p < 0.01 by Student’s *t* test; ***p < 0.001 by Student’s *t* test

To further confirm the suppressive effect of miR-378a on tenogenic differentiation, we transfected miR-378a mimics on TDSCs to investigate their effects during tenogenic differentiation. As shown in Fig. 4a, miR-378a-transfected TDSCs showed significantly decreased collagen production by Sirius Red staining. On tenogenic marker expression, TDSCs transfected with miR-378a showed significantly decreased expression of *Scx*, *Mkx*, *Fmod*, and *Thbs4*, and significantly increased expression of *Mmp3* on day 2 (Fig. 4b). And collagen type 1 and collagen type 3 were also inhibited significantly in addition to other markers in the Tg mice on day 4 (Fig. 4d). These indicated that miR-378a could inhibit tenogenic differentiation. Similarly, *Ywhaz* was also selected as the housekeeping gene when analyzing these data. As shown in Additional file 3: Figure S2C, the result with using *Ywhaz* as the internal control remained consistent with the data using *Rpl13a* as the internal control. We next transfected the TDSCs with anti-miR-378a mimics, to see whether they could reverse the inhibition effects of miR-378a on TDSCs during tenogenic differentiation. Figure 4c shows significantly increased collagen type 1 production in TDSCs transfected with anti-miR-378a. The tenogenic markers of *Scx*, *Mkx*, and *Collagen type 1* were significantly increased in TDSCs transfected with anti-miR-378a, while *Mmp3* and *Thbs4* were inhibited significantly. No significant difference was observed on *Col3a1* and *Fmod* expression between groups. However, when analyzing data using *Ywhaz* as the housekeeping gene, only *Scx* was significantly increased in TDSCs transfected with anti-miR-378a (Additional file 3: Figure S2C & D). The overall results were consistent with the inhibited tenogenic differentiation effects in TDSCs isolated from the Tg mice, which further indicated that miR-378a could inhibit tenogenic differentiation in TDSCs under TGFβ1 induction.

### TGFβ2 was a novel target of miR-378a in TDSCs

**Fig. 5:**
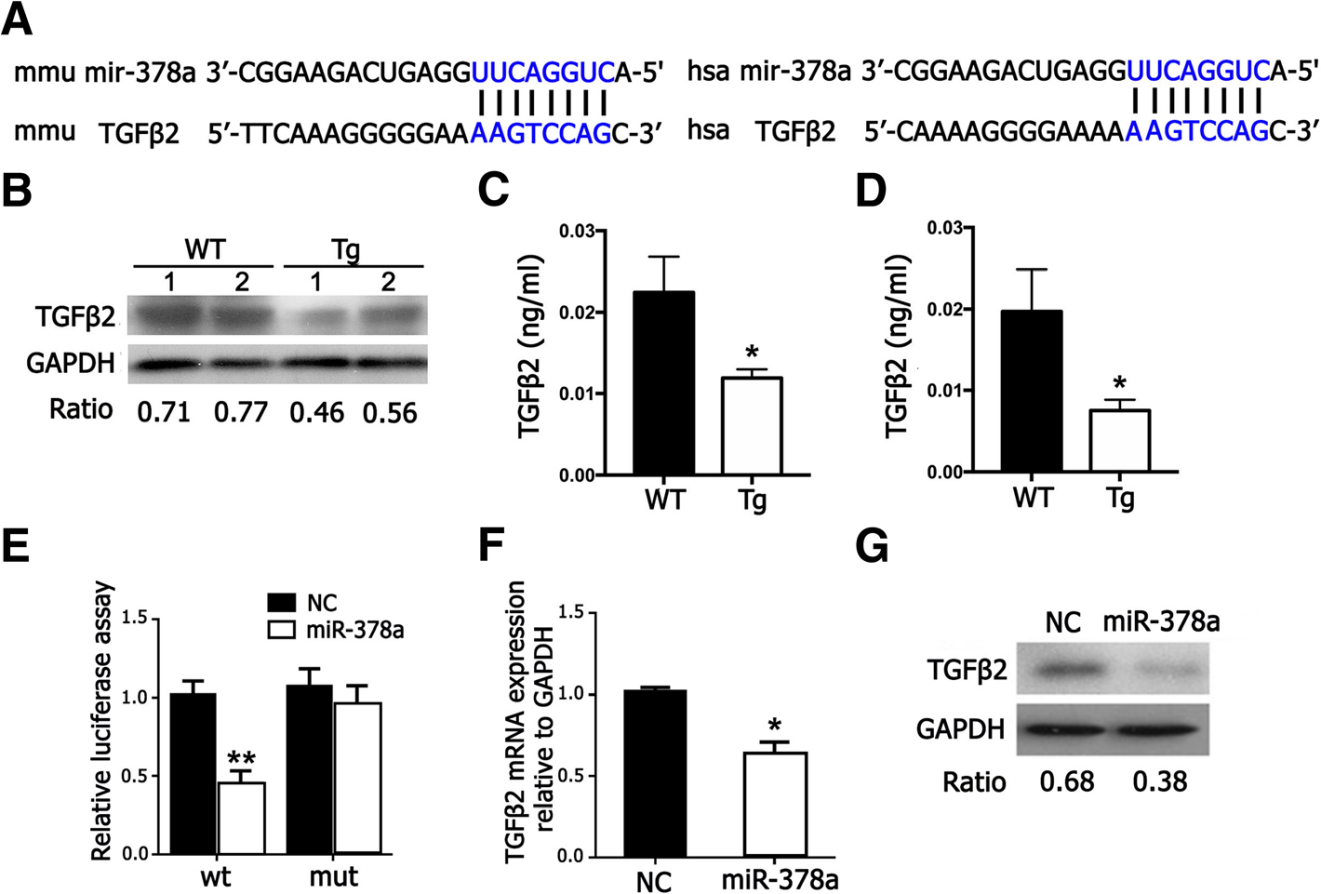
TGFβ2 was a novel target of miR-378a in TDSCs. **a** The predict binding target of TGFβ2 for miR-378a in mouse and human. **b** The expression of TGFβ2 in TDSCs from WT and Tg by western blotting assay. **c** The expression of TGFβ2 in TDSCs from WT and Tg mice by ELISA assay. **d** The expression of TGFβ2 in TDSCs from WT and Tg under TGFβ1 induction by ELISA assay. **e** MiR-378a was co-transfected with the TGFβ2 luciferase reporter into TDSCs, and the luciferase activity were measured. **f**, **g** The expression of TGFβ2 was examined in miR-378a-transfected TDSCs at mRNA and protein levels. *n* = 3; *p < 0.05 by Student’s *t* test; **p < 0.01 by Student’s *t* test

As mentioned before, miRNA exerts its biological role through binding at the 3′UTR region of target mRNA, to negatively regulate the target gene expression. Using the prediction databases (miRBase, miRDB, and TarBase), miR-378a was predicted to have the binding target at TGFβ2 in both human and mouse (Fig. 5a). We first compared the TGFβ2 expression in TDSCs from the WT and Tg mice. As shown in Fig. 5b, c, TGFβ2 expression was decreased in the Tg mice by western blotting and ELISA. Moreover, TGFβ2 was also inhibited in TDSCs from the Tg mice under TGFβ1 induction (Fig. 5d). To further confirm the binding target of miR-378a on TGFβ2, the target sites into the 3′UTR locus of TGFβ2 were inserted into the firefly luciferase reporter, and the luciferase activity was measured. It was showed that miR-378a dramatically suppressed the luciferase activity and that mutations on these binding sites successfully abolished the suppressive effect (Fig. 5e). Furthermore, both the mRNA and proteins levels of TGFβ2 were inhibited in TDSCs transfected with miR-378a (Fig. 5f, g). All the results demonstrated that TGFβ2 was a bona fide target for miR-378a.

## Discussion

Our study indicated the inhibitory role of miR-378a during tenogenic differentiation, especially its suppressive effects on collagen and ECM production both in vivo and in vitro. Mechanically, our results demonstrated that miR-378a could bind at TGFβ2 to downregulate its expression, which further contributes to the inhibition effects during tenogenic differentiation. Targeting at miR-378a could be considered to develop a new potential biomarker or drug target for possible therapeutic approach in clinical practice.

Tendon tissue are mainly made up of collagen type I and surrounding ECM [18]. The injured tendons are always accompanied with altered collagen production, impaired extracellular matrix remodeling, and inflammatory cell infiltration, which lead to the incidence of re-injury [18, 19]. Due to the high prevalence of weight-bearing tendon injuries [20, 21], we selected the patellar tendon to explore the biological role of miR-378a on tendon differentiation and regeneration. The TGFβ pathway is one of the most recognized signaling pathways for tendon development [22]. It has been reported that the *Tgfβ2* and *Tgfβ3* double knockout mice or *Tgfβr2* knockout mice show loss of most tendons [23, 24]. Tenogenic differentiation with TGFβ1 is regarded as the classical method in vitro [22]. In the present study, we found tenogenic differentiation was suppressed in TDSCs derived from the miR-378a Tg mice via abnormal collagen type 1 and ECM (such as Fmod, Mmp3, and Thbs4) production. And a delayed tendon injury healing, with an increased cell number and lower collagen type 1 and Thbs4 production, was also observed in the Tg mice. The observed increased interfibrillar space in tendons from the Tg mice also indicated miR-378a could inhibit ECM production. The mimics in vitro results also confirmed the suppressive effect of miR-378aon tenogenic differentiation. As mentioned before, collagen type 1 is the main composition of tendon tissue. During the development of tendons, it has been shown that the TGFβ-Scx pathway plays a critical role in the initial differentiation of tendons, followed by the TGFβ-Mkx pathway playing an essential role at the tendon maturation stage [25]. In our study, decreased collagen type 1 production can be observed both in vivo and in vitro, which may have the relationship with the suppressive effects of miR-378a on *Scx* and *Mkx*. Moreover, the inhibitory role of miR-378a on *Scx* and *Mkx* also potentially indicates that miR-378a suppresses tenogenic differentiation via TGFβ signaling. Overall, our results demonstrated that miR-378a could inhibit tenogenic differentiation by suppressing collagen type 1 and ECM production and also impair tendon injury healing*.*

MiRNA exert its regulatory role on gene expression through binding the 3′-untranslated region (UTR) of their target mRNA leading to translational repression or mRNA degradation. To find the binding target of miR-378a during tenogenic differentiation, the miRNA biological databases of miRBase, miRDB, and TarBase were used in our study. The results indicated that miR-378a has the binding target at TGFβ2 both in mouse and human. To examine whether TGFβ2 expression was influenced by miR-378a during tenogenic differentiation, we applied western blot and luciferase assay. Our results showed that TGFβ2 expression was inhibited in TDSCs from the Tg mice at both mRNA and protein levels. By luciferase assay, miR-378a could inhibit the activity of the luciferase reporter harboring TGFβ2 gene, while its inhibitory effect was abolished when the binding sites on the TGFβ2 gene were mutated. This strongly indicated that TGFβ2 was targeted by miR-378a during tenogenic differentiation. Interestingly, Yu et al. also showed that miR-378a could target TGFβ2- in TGFβ1-treated hepatic stellate cells [26]. Of course, it remains a possibility that there may have been other targets of miR-378a [27]. However, as TGF-β signaling is essential for tenogenic differentiation, we think TGF-β2 would be the main target of miR-378a during tenogenic differentiation. For the possibility of any other potential targets, it is also worth to be studied in the future.

## Conclusion

Taken together, our results indicated that miR-378a could suppress tenogenic differentiation, inhibit ECM production, and impair tendon injury healing. TGFβ2 was identified as a novel target, and its expression was suppressed by miR-378a. Therefore, miR-378a suppressed tenogenesis via reducing TGFβ2. Targeting at miR-378a could be considered to develop a new potential biomarker or drug target for a possible therapeutic approach in clinical practice.

## Additional files

Additional file 1: **Table S1.** Mouse primers for qRT-PCR. (DOCX 15 kb)

Additional file 2: **Figure S1.** Size distribution of fibril diameter between WT and Tg mice. (JPG 136 kb)

Additional file 3: **Figure S2.** Tenogenic differentiation analysis using *Ywhaz* as a housekeeping gene. (A) Tenogenic marker expression during differentiation in TDSCs derived from WT and miR-378a Tg mice at day 2 and (B) day 4. (C) Tenogenic marker expression during differentiation in TDSCs transfected with miR-378a mimics and (D) anti-miR-378a mimics. *n* = 3; *, *p* < 0.05 by Student’s *t* test. (JPG 798 kb)

## Abbreviations

Col1A1: Collagen type I α 1 chain
Col3A1: Collagen type III α 1 chain
ECM: Extracellular matrix
Fmod: Fibromodulin
GAPDH: Glyceraldehyde 3-phosphate dehydrogenase
H&E: Hematoxylin and eosin
miRNA: MicroRNA
miR-378a: miR-378a-3p
Mkx: Mohawk
Mmp3: Matrix metalloproteinase-3
Rpl13a: Ribosomal protein 13a
Scx: Scleraxis
SEM: Scanning electron microscopy
TDSCs: Tendon-derived stem cells
TEM: Transmission electron microscopy
Tg: Transgenic
TGF: Transforming growth factor
Thbs4: Thrombospondin 4
UTR: 3′-Untranslated region
WT: Wild type
