# Supplementary material for: MiR-378a suppresses tenogenic differentiation and tendon repair by targeting at TGF-β2
Source: Stem Cell Res Ther. 2019 Mar 29;10:108. doi: 10.1186/s13287-019-1216-y (PMC6440014; doi:10.1186/s13287-019-1216-y)
Supplement: Supplementary file 1 — Table S1. Mouse primers for qRT-PCR. (DOCX 15 kb) [file 13287_2019_1216_MOESM1_ESM.docx]

**Table S1** Mouse primers for qRT-PCR

| **gene** | **Forward primers (5' to 3')** | **Reverse primers (5' to 3')** |
| --- | --- | --- |
| *Scx* | CCTTCTGCCTCAGCAACCAG | GGTCCAAAGTGGGGCTCTCCGTGACT |
| *Mkx* | AGTAAAGACAGTCAAGCTGCCACTG | TCCTGGCCACTCTAGAAGCG |
| *Col1A1* | TGGAGAGAGCATGACCGATG | GAGCCCTCGCTTCCGTACT |
| *Col3A1* | CTAAAATTCTGCCACCCCGAA | AGGATCAACCCAGTATTCTCCACTC |
| *Fmod* | CTTTACCTCCAGGGCAACAGG | GCTTGATCTCGTTCCCATCCAG |
| *MMP3* | CAGACTTGTCCCGTTTCCAT | GGTGCTGACTGCATCAAAGA |
| *Thbs4* | GCTCTGTAGAACTCTACCTGGACT | TCTTCCAAGAAGTCCTGGGGCTTC |
| *Tgfβ2* | CCACATCTCCTGCTAATGTTG | AGTAGGCAGCATCCAAAGC |
| *Rpl13a* | AGGGGCAGGTTCTGGTATTG | TGTTGATGCCTTCACAGCGT |
| *Ywhaz* | GAAAAGTTCTTGATCCCCAATGC | TGTGACTGGTCCACAATTCCTT |
| *Gapdh* | CCTGGTCACCAGGGCTGC | CGCTCCTGGAAGATGGTGATG |
