# Supplementary figures and images for: MiR-378a suppresses tenogenic differentiation and tendon repair by targeting at TGF-β2
Source: Stem Cell Res Ther. 2019 Mar 29;10:108. doi: 10.1186/s13287-019-1216-y (PMC6440014; doi:10.1186/s13287-019-1216-y)

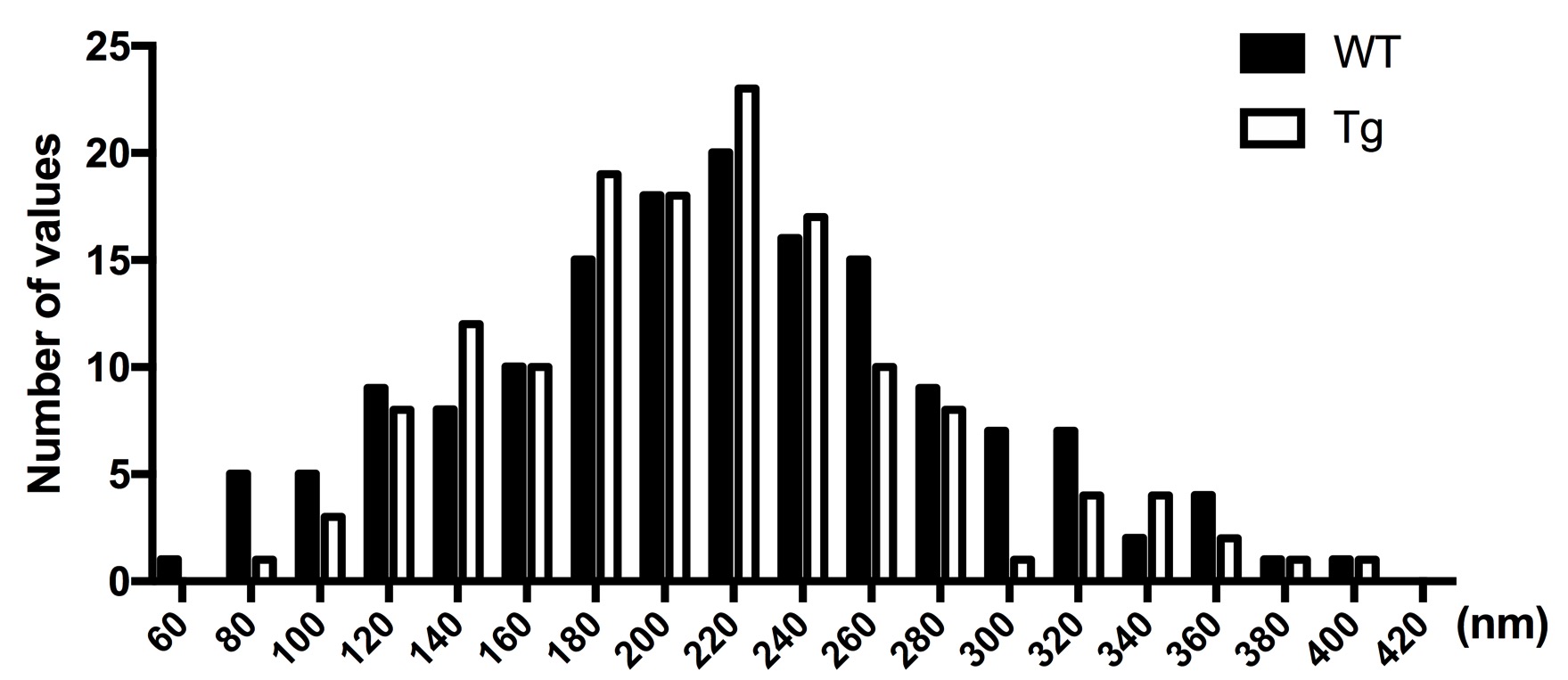

Supplement: Supplementary file 2 — Figure S1. Size distribution of fibril diameter between WT and Tg mice. (JPG 136 kb) [file 13287_2019_1216_MOESM2_ESM.jpg]

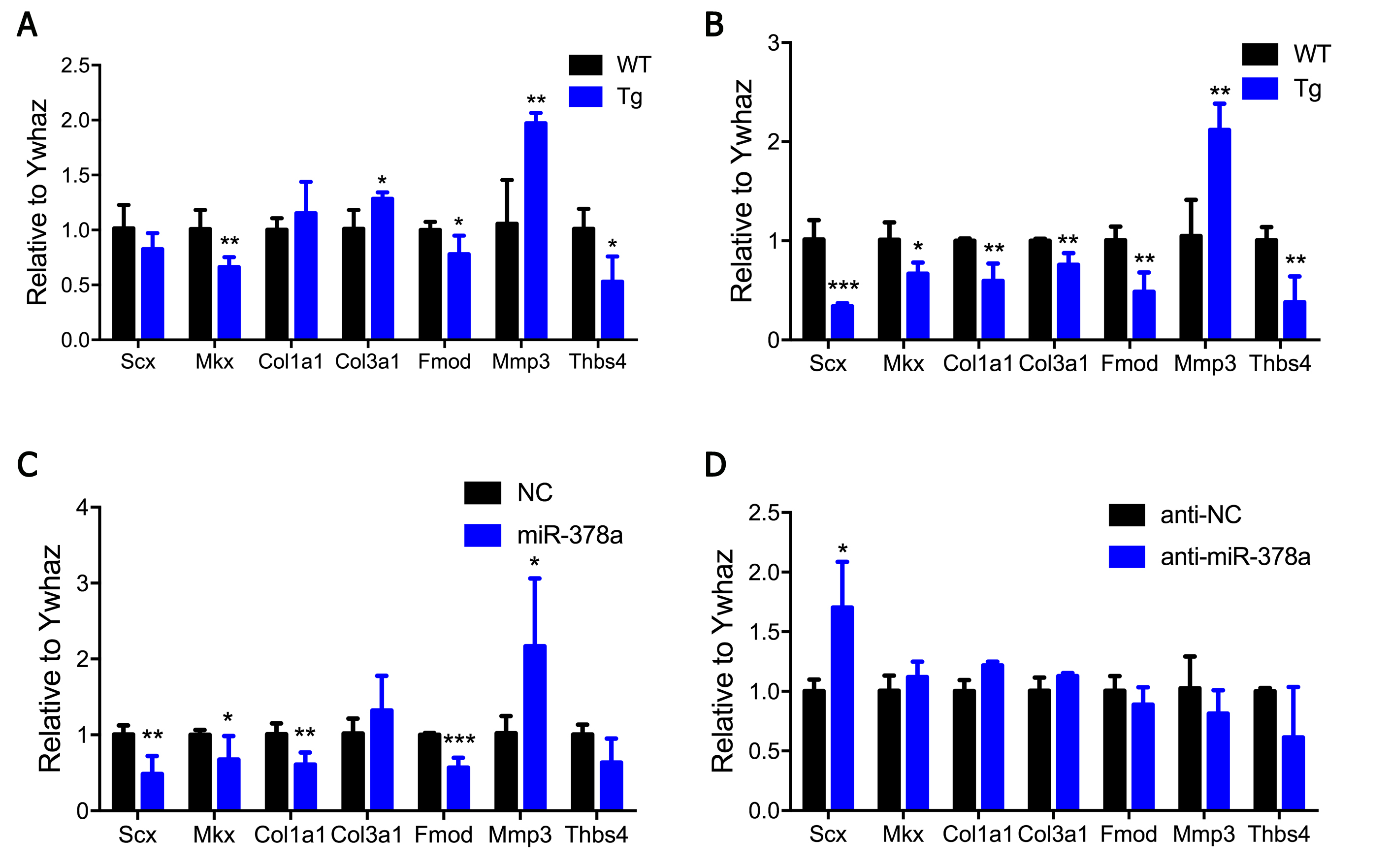

Supplement: Supplementary file 3 — Figure S2. Tenogenic differentiation analysis using Ywhaz as a housekeeping gene. (A) Tenogenic marker expression during differentiation in TDSCs derived from WT and miR-378a Tg mice at day 2 and (B) day 4. (C) Tenogenic marker expression during differentiation in TDSCs transfected with miR-378a mimics and (D) anti-miR-378a mimics. n = 3; *, p < 0.05 by Student’s t test. (JPG 798 kb) [file 13287_2019_1216_MOESM3_ESM.jpg]
